# Social Hazards as Manifested Workplace Discrimination and Health (Vietnamese and Ukrainian Female and Male Migrants in Czechia)

**DOI:** 10.3390/ijerph14101207

**Published:** 2017-10-10

**Authors:** Dušan Drbohlav, Dagmar Dzúrová

**Affiliations:** Department of Social Geography and Regional Development, Faculty of Science, Charles University, Albertov 6, 128 43 Prague 2, Czech Republic; dusan.drbohlav@natur.cuni.cz

**Keywords:** self-reported health, workplace, social hazards, discrimination, Vietnamese and Ukrainian immigrants, Czechia, questionnaire survey, logistic regression

## Abstract

Social hazards as one of the dimensions of workplace discrimination are a potential social determinant of health inequalities. The aim of this study was to investigate relations between self-reported health and social hazard characteristics (defined as—discrimination as such, violence or threat of violence, time pressure or work overload and risk of accident) among Vietnamese and Ukrainian migrants (males and females) in Czechia by age, education level and marital status. This study is based on data from a survey of 669 immigrants in Czechia in 2013. Logistic regression analysis indicates that the given independent variables (given social hazards and socio-demographic characteristics), as predictors of a quality of self-reported health are more important for immigrant females than for males, irrespective of citizenship, albeit only for some of them and to differing extents. We found out that being exposed to the selected social hazards in the workplace leads to worsening self-rated health, especially for females. On the other hand, there was no statistically significant relationship found between poor self-rated health and discrimination as such. Reality calls for more research and, consequently, better policies and practices in the field of health inequalities.

## 1. Introduction and Background

Out of all possible human activites, work is a key determinant of man’s health. Around the globe, work is a fundamental human activity that consumes many of people's waking hours.

Work may be, and sometimes is, accompanied by discrimination. Workplace discrimination takes several forms (from e.g., violence through sexual harassment to work overload) and is a socially determined factor that can potentially make an impact on health inequalities. The nature of the association between health status and work discrimination has repeatedly been demonstrated by various populations in various contexts (see more below). Moreover, the probability of being discriminated against is higher when besides other often vulnerable population groups—international migrants, namely those, who take manually demanding, low-skilled jobs, come into the play [1].

This section will set the scene for the topic of s association between discrimination, health and migration. This theme is elaborated upon when specifically investigating ethnic differences among two of the most common immigrant groups in Czechia (Ukrainian and Vietnamese) in self-reported health and social hazards/risk factors in the workplace.

Below, we introduce both discrimination and migration issues vis-a-vis health matters as to how these relationships are reflected in the scientific world. The contribution is further structured in a standard way: state-of-art in Czechia comes before methods, then, analysis, results, discussion and conclusions follow.

### 1.1. Discrimination and Health

There are many examples suggesting that people who perceive more interpersonal discrimination directed at themselves or other members of their group are at greater risk of a worse health status. Or, in other words, perceived discrimination has a significant negative effect on both mental [2,3,4,5,6,7,8,9] and physical health [2,3,4,5,6,7,8,9,10]. It has also often been shown that such an effect tends to be manifested more in relation to mental health vis-a-vis physical one—see [4,6,7,9,11]. More specifically, various studies clearly show how discrimination is strongly related to blood pressure, hypertension, and cardiovascular conditions and reactivity (see examples in [12]). Moreover, many objective clinical disease outcomes are cited in relation to discrimination (see [6])—such as incident breast cancer, incident asthma, coronary artery calcification, visceral fat or inflammation among others. Furthermore, one of the studies on discrimination have suggested that stressors can lead to the onset and aggravation of chronic pain and headaches (see [12]). Although, in research activities, racial/ethnic discrimination is very often a synonym for discrimination as such, other reasons for discrimination are studied too, such as gender or sex, age, height or weight, sexual orientation, income, education, homelessness [12,13,14] and also migratory status [15].

It has been indicated that “research suggests that racial and non-racial forms of interpersonal discrimination have similar associations with health” ([6], p. 429, see also [16], p. 941). Nevertheless, we do not know the reasons for such associations and the mechanisms which lie behind it. Research has shown that perceptions of certain types of discrimination is associated with an increased likelihood of perceiving other kinds of discrimination (for example, individuals with more severe lifetime substance use problems were significantly more likely to report discrimination due to homelessness/poverty and race/ethnicity/skin colour in healthcare setting—[14]). In this regard, also Grollman’s analysis (2014) shows us that “multiply disadvantaged adults are more likely to experience major depression, poor physical health, and functional limitations than their singly disadvantaged and privileged counterparts” ([17], p. 3) (see also [6,11]). However, we do not know well, yet, how multiple types of discrimination or other unfair treatments combine to affect health [6].

Krieger (2012)—in her ecosocial theory of disease distribution, which deals with who and what drives social inequalities in health—also defines important areas of hazards that may enter discrimination at various levels and dimensions: (1) socioeconomic deprivation, (2) occupational hazards (i.e., chemicals, dusts, fumes, and ergonomic strain), (3) social hazards (i.e., racial discrimination, workplace abuse, and sexual harassment at work), and (4) relationship hazards. There is a need to examine discrimination and health association in a complex way, in conjunction with other stressors related to societal [6], psychological and environmental disadvantages/stressors [18]. Due to the complex and very complicated nature of transmission of stressors and their transformation into health outcomes (1) discrimination, (2) threat, (3) fear, anger, denial, etc., (4) physiological responses: cardiovascular, endocrine, neurological, immune, etc. (5) health outcome—see in [13], to find the right causalities within the studied relationships is a very difficult task.

### 1.2. International Migration/Migrants and Health

To be a migrant and to have an immigration status means to have a disadvantage in the society of the destination country—they create one possibly marginalized group (but often except those who are highly skilled in important positions) (e.g., [1,15,19]). Due to migration flows which also contribute to diversifying population structures in many immigration countries in the world [20,21], governments and societies have to face public health challenges in coping with health issues of migrants [22,23,24]. (Currently, foreign labour force represents in many developed countries quite important segments—for example, it is in the USA 18%, the United Kingdom 16%, Austria 19%, Belgium 15%, Germany 16% or Sweden 18% (data relates to foreign-born in total employment in 2015—see [25]). Despite disturbing role of economic crises, the respective shares do not seem to decrease in a long-term perspective, rather the other way round).

Knowledge about SRH can help us better understand the health status and needs of migrants. Regardless of their migrant status, international migrants, mainly those with manually demanding work, can be at risk of poor physical and mental health since they are often excluded from legal frameworks relating to occupational health and safety [26]. Migrants, as compared to the remaining majority of individual populations, tend to be more discriminated against in various spheres including work relations and conditions [27]. Discrimination against immigrants is often omnipresent. Agudelo-Suárez et al. [28] describe well how complex immigrant discrimination is in their qualitative study in Spain: “The discourses provided by the immigrant population that took part in this study reveal a perception of discrimination through feelings of having experienced racism and isolation, and in relation to their working and social environment, through the types of contracts offered, as well as factors such as ethnic background, nationality and culture. Other aspects related with institutions and economic, legal and political processes were also identified. They recognise that discrimination and its different manifestations have an impact on the quality of life of the affected groups and therefore on their health and well-being” [28] (p. 1872). This picture might be found in many developed countries with a huge pool of foreign labour especially in their secondary labour markets.

Deprivation and all other hazards (see e.g., Krieger’s theory above—[13,16]) may constitute risk factors contributing to poorer health of immigrants. As for research results on health of migrants, many studies have reached the conclusion that most migrants are disadvantaged as compared to the majority of the population, even after controlling for age, gender, and socioeconomic factors (see e.g., in [10,23,29,30,31]). Nevertheless, some other results of research activities may not identify the same significant differences (e.g., [32]). What seems to be often confirmed is that health of immigrants deteriorates in a host country as time passes, thereby suppressing the “healthy migrant effect”. It is explained especially via positive self-selection of the health of immigrants and positive selection by the host countries (e.g., [23,33,34,35]).

The perception of discrimination and poor health is crucial in shaping the migration process. Accordingly, the occupational health of migrants—with exception of those highly qualified holding important positions—is burdened with many occupational and social hazards [13,16] and also tends to be worse than that of non-migrants [36,37]; of course occupation and industry may affect the association between discrimination and health, too. Migrants are at risk of not receiving the same level of health care due to a combination of several factors including legal status, language and cultural barriers, whilst mostly occupying low-qualified and high-risk jobs [38]. This is even more complicated since international migration movements bring about a new diversity or even “super-diversity” [21] which becomes typical of many populations in many countries through the whole world.

On top of this, as the current refugee crisis shows us, in addition to those who move voluntarily and whose movements are unambiguously triggered by economically driven motives, many migrants are now forced to leave their home countries, regions and localities [20,39]. This means their integration into host societies and, possibly, host labour markets is even more difficult and problematic (including relevant health issues) [40]. All these realities are giving rise to vociferous calls for more intensive and sophisticated research into health issues of immigrants in the broadest sense of these words. In this context, aspects related to discrimination, unfair treatment, self-rated health and gender dimension are undoubtedly some of the most prominent topics.

When summarizing research activities in the field of “discrimination and health” with special regard to immigrants or diverse domestic population groups, most of the studies to date were published in the US (mostly looking at African Americans, Latinos or other immigrant groups), be it conceptual [13,16], methodological/methodical [41] or “summarizing meta-reports” (like e.g., [6,7] or [8] or other various studies—see e.g., [9,11,12,42,43,44,45,46,47]). Nevertheless, some empirical studies have also been done in Western, Northern and Southern Europe—e.g., [5,19,28,31,37,48]. We have, however, found no similar study in Central/Eastern Europe, where research into these issues, particularly in post-communist countries, is very underdeveloped. One of the countries with the fastest growing economic migration (at least until 2008, when the global economic crisis started), especially from third-countries (migrants coming from outside the EU) is Czechia—thus providing an ideal laboratory for researching the issues outlined above.

### 1.3. State-of-Art in Czechia

Czechia, or rather the Czech Republic within Czechoslovakia (between 1948 and 1989) was a homogenous society with very small numbers of “standard international immigrants” (see [49]). Since the Velvet Revolution (in 1989), a total transformation of the society—from a socialist/communist regime to a democratic, parliamentary system based on a free market economy —has started in Czechia. This transformation process, along with ongoing globalization and the shift towards a post-modern society, has brought significant inflows of immigrants [50]. Over time, the number of immigrants grew and reached slightly more than 400,000 legally registered immigrants in 2008. Since then, this figure has stayed more or less the same and stood at 449,000 foreigners at the very end of 2014 (out of this number 185,000 immigrants came from the EU [51]). Ukrainians, Slovaks, Vietnamese, Russians and Poles are among the most important immigrant groups [50].

In the course of time, some research initiatives also touched on immigrants’ health and closely related issues [52,53,54,55]. However, for example, research devoted to analysing the role of gender within the migration process (not specifically health issues) is rare in the Czech context—see only two examples representing exceptions to the trend—[56,57].

It is well-known that the health of immigrants in Czechia and related health services that are used by immigrants in the country are underestimated issues and much less researched than is necessary. This concerns also more specific aspects such as studying differences in the health of migrants broken down by various characteristics such as ethnicity, age, education, length of stay, etc. In any case, currently migratory issues including the state of physical and mental health of migrants are important challenges for policy and policy-makers to balance disparities in labour and social rights. Of course, it is not only a Czech problem, in fact, all European countries where migrants represent important minority groups have to adjust to growing diversity while also adopting specific migrant health policies [58]. During the last two decades the number of insecure jobs in Czechia has increased significantly along with the number of immigrants who are active on the Czech labour market [49,59]. Despite this, in harmony with the situation in other CEE countries (as already mentioned), there is a very superficial knowledge of the health of migrants in Czechia, not to mention the relationships to their life style, work or factors that stand behind and modify the state of their health. Not surprisingly, Czech policymakers are not familiar with the milieu in which migrants live and work. All these gaps—insufficient research results and information/data, and the lacking interest of politicians—have made it very difficult to successfully combat discrimination and unfair treatment of migrant populations, both in Czechia and at the EU level.

Despite the above criticism, recent studies on migration and health issues in Czechia (focused primarily on health insurance and self-rated health and discrimination) have provided several important results:

Significant inequalities in access to healthcare were found in a context characterised by the exclusion of some categories of long-term immigrants from the public healthcare systems [12]. This study of Ukrainian immigrants in Czechia showed that access by Ukrainian immigrants with long-term visas to most types of healthcare, including primary care, is substantially lower, as compared not only to native Czechs, but also to fellow nationals with a permanent residence permit [60]. Similarly, a survey of Ukrainian and Vietnamese immigrants in Czechia showed us that among immigrants entitled to Czech public health insurance due to permanent residency/asylum, 30% were not in the public health insurance system, and of those entitled by their employment status, 50% were not in the system [61]. Migrants with poor knowledge of the Czech language are more likely to remain excluded from the system of public health insurance [61]. Finally, there is an analysis of Ukrainian immigrants in Czechia (versus the Czech population) which concentrated on the relationship between immigrants’ self-reported/rated health (SRH) and their perceived working conditions materialized via discrimination [62]. In this article discrimination was assessed along with other exposures to risks in the workplace. By contrast, in this contribution we approach this issue in a different way, making new use of a concept of social hazards see [13,16].

## 2. Methods

### 2.1. Aim of This Study

The aim of this study is to investigate ethnic differences among two of the most common immigrant groups in Czechia (Ukrainian and Vietnamese) in self-reported health and social hazards/risk factors in the workplace. Respondents were legally staying foreigners who have lived and worked in Czechia for at least one year. Our goal is to ascertain whether there is an association between poorer health and the social hazards and if yes, what type or types of social hazards come into play and what role they play.

We perceive (and work with) social hazard categories to some extent in line with Krieger’ ecosocial theory (2012) as one of the manifestations of discrimination. While modifying Krieger’s social hazards dimension categories at workplace we used the following characteristics throughout our analysis: discrimination (as such), violence or threat of violence, time pressure or work overload and risk of accident (see more below). As far as migration characteristics are concerned, we relied on answers to our questions asked via a questionnaire survey to selected Vietnamese and Ukrainian migrants, who have been staying as long-term or permanent status holders and working mostly in rather low-level jobs in Czechia. As for health status, we applied a common way as to how to measure a person’s health via self-rated health (SRH). SRH is generally considered a “a strong and independent predictor of mortality and has good test-retest reliability” [63] (p. 151).

The targeted immigrant population (see below) are at the intersection of two or more disadvantaged statuses—see e.g., low-income Ukrainian/Vietnamese women (more on the concept of intersectionality in [17]). If being disadvantaged within one sphere, then, other unfair treatments may easily accumulate—see multiple forms of discrimination also in [13,16]. Based on these concepts, hence, we can also deduce that individuals who report more types of social hazards are also more likely to report poorer health.

### 2.2. Target Groups

Two immigrant groups we are dealing with were Vietnamese and Ukrainians.

Vietnamese started arriving in Czechia (within the former Czechoslovakia) in as early as the 1950s under the so-called international aid among socialist/communist countries (political and economic mutual support). Apart from students who stayed in the biggest cities, they spread through the whole country while working in many factories as temporary workers or trainees for some years. During the 1980s, just before the Velvet Revolution, their numbers oscillated between 20,000 and 30,000 (see [64,65]). The inflow of Vietnamese citizens to Czechia/Czechoslovakia started decreasing in 1986 when the “Doi Moi”, economic reforms started in Vietnam and it stopped after the Velvet Revolution in Czechoslovakia in 1989. Since then, a new era of Vietnamese migration in Czechia has started. Due to their experience and established social networks and relevant contacts, new Vietnamese including migrants’ family members started coming [66]. Their numbers gradually grew and they filled a gap on the Czech market chiefly in the role of small entrepreneurs (now some 90% of economically active Vietnamese) currently involved in wholesale, but mostly retail- while running food stores (small family-operated shops) or, newly, also nail studios. The Vietnamese population is characterized by strong intracommunity social networks [67]. As of 31 December 2014, 57,000 Vietnamese legally reside in Czechia [50,51], making up the third largest Vietnamese immigrant group in Europe, after France and Germany.

Ukraine immigration dates back to the era of the Austro-Hungarian Monarchy. Moreover, Zakarpathian Ukraine was a part of independent Czechoslovakia during the 1920s and 1930s. Czechoslovakia also accepted many Ukrainians who escaped from the Soviet Union mainly in the aftermath of the October Revolution. After the Velvet Revolution, since the very beginning of the 1990s, Ukrainians started coming to Czechia as a temporary, circular labour force [49,68]. Czechia became an attractive destination for Ukrainians since they could find common Slavic roots and culture, and to some extent a similar language, and also it was “not so far to get there”. As a result, masses of migrants came and filled gaps on the Czech labour market where Ukrainian workers were and are strongly overrepresented in the lowest-skilled sector (the “secondary one” within the dual labour market concept) (e.g., [69]). Hence, one can find Ukrainian workers (mainly employees) mostly in construction (as an auxiliary labour force), but also in some industrial sectors, such as agriculture and services (including domestic workers) and as undocumented labour force as well [50]. Ukrainians represent the most important immigrant group in the country (except for a specific Slovak group) with 104,000 legally resident migrants (as of 31 December 2014) (see e.g., [70]). Whereas Ukrainians “traditionally” represented a “transnational” migrant group (see [71,72]) where migrants usually circulate between Czechia and Ukraine (see [67]), now, there is an increasing trend among Ukrainian migrants to more often settle in Czechia—currently 71 percent are permanent residence holders (see [51]).

It is this “third-country” immigrant group active in a “dual labour market” that might be the most vulnerable to discrimination and unfair treatment in many various ways among all of those who are economically active in Czechia.

### 2.3. Sample Design and Size and Survey Instrument

We applied a quantitative approach using a questionnaire survey as our main research tool (see similar “philosophy” (for example, in [73])). For the purpose of this study we used data from the survey that targeted Ukrainian and Vietnamese migrants who were living and working in Czechia. Whereas the survey mainly targeted patterns of internal migration and related life style characteristics, the questionnaire survey also included some common characteristics and questions related to ascertaining migrants’ SHR and issues of social hazards in the workplace. Thus, we got a robust sample, although at the expense of losing the possibility to include more specific characteristics in our analysis.

Selection of the respondents was done via a quota sampling method using the 2011 Czech Census data (sex, age and region of migrant’s residence were selected as quota characteristics). Only immigrants having Ukrainian or Vietnamese citizenship, those who have stayed in Czechia at least for 1 year and were older than 15 qualified to enter the survey. As already indicated, immigrants migrating more intensively/frequently inside Czechia were intentionally overrepresented in the sample.

The survey was carried out among 912 respondents (445 Vietnamese and 467 Ukrainians) between March and May 2013 (the response rate was about 80 %). The original file contained respondents aged 15—65 years. In the first step, for the purpose of analysing social hazards in the workplace we used only the file of respondents aged 18–62 years. The reason was that we wanted our results to be comparable with those in previous studies (where respondents were also in the age group 18–62 years (see [62]). In the second stage, we excluded those respondents from the original set for whom we lacked data on social hazards in the workplace, giving us a smaller sample of 669 immigrants (see more below).

To make our task easier, when researching the complicated relations and factors behind SRH and the risk of social hazards in the workplace, we worked with several very important but only basic factors: gender, age, educational level and marital status. The social hazards of immigrants at the workplace are based on Krieger’s ecosocial theory where she defines social hazards explicitly as racial discrimination, workplace abuse and sexual harassment at work. In accordance with the above while partly modifying the given social hazard categories, we used the following social risks at the workplace for our analysis: discrimination at workplace, violence or threat of violence, time pressure or overload of work and risk of accident (see inspiration in [74])—more details see in part Dependent and Independent variables. To our knowledge, this is one of the first studies in the region with the sufficient data to investigate the relationship between self-rated health, various social hazards in the workplace and selected socio-demographic indicators for immigrants from Ukraine and Vietnam (broken down by sex).

### 2.4. Survey Administration

The survey was carried out by the interviewers’ network of the Public Opinion Research Centre, which is affiliated with the Institute of Sociology of the Czech Academy of Sciences. The interviewers were to find appropriate respondents (they gained information about locations where immigrants may possibly be found), to distribute questionnaires and then to collect them. The respondents filled in the questionnaire (in self-administered mode) in their mother language. This task lasted on average about 45 min.

### 2.5. Dependent and Independent Variables

Perceived health was the dependent variable we were interested in. The socio-demographic variables: age, education, marital status and selected characteristics of social hazards in the workplace were included in the analysis as independent variables.

The dependent variable—perceived health (Self-rated health, SHR) has been one of the most frequently used variables in health research (see e.g., [11,31,53,63]). SRH was measured in our survey on a 5-point scale ranging from very good to very poor (the question was: “How is your health in general?”) and was dichotomized as “Good” (Good/very good) or “Not good” (Poor/poor/not good-not bad).

The independent variable connected with social hazards in the workplace was determined in our survey by answers to the four questions: “At your workplace, to what extent are you exposed to: (a) “Discrimination (as such; D); (b) Violence or threat of violence (V); (c) Time pressure or overload of work (T); (d) Risk of accident” (A)? Possible answers were: Not exposed, occasionally/slightly, repeatedly, regularly/strongly, don't know or refusal and was dichotomised as “Yes, exposed” (If he/she was exposed) or “No exposure”. Apart from “discriminations as such”, this categorization was inspired by Lucarelli and Boschetto [74], who applied a wide mosaic of social hazard characteristics (called risk factors of health and safety at work) within their survey in Italy (Vulnerable Workers and the LFS Ad Hoc Module).

In the first step, these measures were used to determine the prevalence of social hazards.

Whereas discrimination as such is analysed separately, we created a new variable—“multiple forms of social hazards” where three selected dichotomized characteristics of social hazards were counted up. A multinomial variable of multiple forms of social hazards was categorized into 3 levels: 0, 1, and 2 (0—not exposed; 1—indicating those with one type of social hazards; 2—those with two or three types of social hazards).

Other basic socio-demographic independent variables entered the analysis in the following form: First, education was categorized into three groups: Primary-basic + secondary without graduation; Secondary − secondary with graduation; University − post-secondary education + university. Second, marital status was categorized into the two categories—married and others. Third, the variable of age was used as an aggregated variable in the first descriptive analysis and then as a continuous variable (in the models).

### 2.6. Analysis

Within this study several research phases of quantitative analysis were done:

(1) Descriptive statistics were used to uncover basic patterns as to the SRH of the two citizenship groups (by sex) by sociodemographic characteristics and selected types of the social hazards in the workplace (see the main sample characteristics—Table 1).

(2) The risk of less than good health was estimated by binary logistic regression, the dependent variable was SRH status (1—not good health and 0—good health), and socio-demographic (age, education and marital status) and social hazards in the workplace characteristics (either discrimination or multiple forms of social hazards) entered the analysis as four independent variables. Fully adjusted odds ratios (OR) for not good SRH were estimated in the eight models, separately for males and females for immigrants from Ukraine and Vietnam, for discrimination and for multiple forms of social hazards thereby identifying the determinants of not good SRH. Table 2 presents fully adjusted odds ratios as outputs of logistic regression models within 95% confidence limits. Significant parameter estimates (odds ratios) are shaded.

All analyses were carried out via using the SPSS (IBM SPSS Inc., Chicago, IL, USA) statistical packages. The use of the data was in accordance with the statutory obligations to protect confidentiality. Individuals cannot be identified from the data provided for analysis.

## 3. Results

Table 1 shows the characteristics of the sample and distribution of the variables. A total of 669 immigrants (aged 18–62 years) were included in the analysis, 366 (55%) of them came from Ukraine while 303 (45%) were Vietnamese. As far as socio-demographic characteristics are concerned, 394 males (58.9%), and 275 females were included (41.1%). More than half of the sample population were married (55 % of immigrants). In addition, 32 % of immigrants were younger than 30 years, 27 % were aged 31–40 years, whereas 41 % were older; 50% of immigrants had completed only primary education. By contrast, the highest proportion of immigrants with the highest level of education was found among females from Ukraine (23%).

Table 1 (see below) provides a description of the sample according to the age groups, educational level, marital status and four selected types of social hazards at work (discrimination as such, violence or threat of violence, time pressure or work overload and risk of accident), age groups, education level, marital status and workplace exposition of the four compared samples (males from Ukraine, males from Vietnam, females from Ukraine and females from Vietnam). Almost 40 % (36.6%) respondents of the whole sample were exposed to discrimination at work. The highest prevalence of discrimination is linked to Vietnamese males (43.5%) in contrast to Vietnamese females where the prevalence is the lowest (31.0%).

Out of the selected social risks, exposure to time pressure or work overload was found to be by far the most important (52.8%). As for the sex, Ukrainian females were more exposed to it (57.0%) as compared to their Vietnamese counterparts (45.2%).

The second part of Table 1 (right side) provides a description of the prevalence rate of not good SRH. The perception of not good health was reported by on average 16.1% of the sample, while females from Vietnam perceived their health most negatively (22.2%). Results confirmed that poor health is common among older adults, the values increase with age in all four samples with only one exception—Vietnamese females between 31 and 40 declared slightly better SRH than those between 18 and 30. There is an interesting pattern regarding the educational level. The perception of the quality of health was worst among the most educated migrants (more than one quarter—28.4%). More specifically, especially Vietnamese females (53.3%), but also Ukrainian females (38.2%) declared poorer SRH.

Moreover, rather poor SRH was typical of married people in the both immigrant groups of both sexes as compared to others (single, widowed or divorced).

Except for Vietnamese males it has been shown that worsening parameters of discrimination as such contribute to deteriorating SRH too. Whereas perceived discrimination at the workplace was highest among the group of Vietnamese males (43.5%), the prevalence of poor health for this group of migrants exposed to discrimination was the lowest (10.4%). Paradoxically, quite opposite results were found with Vietnamese females (31.0% versus 30.8%).

Table 2 summarizes the results of eight separate binary logistic models of associations between not good SRH and self-reported social hazards in the workplace (discrimination as such and “multiple forms” of other social hazards) and age, education and marital status (for Ukrainians, Vietnamese, females and males). Significant parameter estimates (Adj. Odds ratios = OR) are shaded.

**(A) Discrimination in 4 Models:**

Based on assessing ORs in all four models no statistically significant relationship between poor SRH and discrimination in the workplace was found. Nevertheless, the highest OR of the discrimination was found for Vietnamese females—this means that the probability of poor SRH was almost three times higher for the females who were discriminated against (similarly, more than twice the level of poor SRH was indicatedd for Ukrainian males).

As for the marital status, in all four models married immigrants always had higher OR than 1, the reference category (between 3.4 and 1.6). This highest OR = 3.381 pertains to Vietnamese females.

The variable describing educational level was detected as statistically significant in relation to poor SRH only among females. OR for lower educational categories compared to the category of university level (OR= 1 as a referent category) is well below OR= 1. It means that the lower the educational level, the better the perceived SRH.

**(B) Multiple Forms of Social Hazards in 4 Models**

The variable “multiple forms of social hazards at work” (composed of the three selected social hazard characteristics—violence or threat of violence, time pressure or overload of work and risk of accident) is statistically significantly linked to OR of poor SRH. First, this conclusion is much more valid for females than males and it can be well documented for the sub-group of Ukrainian females. When perceiving one of the social hazards then the probability of poor SRH is more than 4.5 times higher compared to those who are not exposed to the selected social hazards (OR = 4.541, 95% CI = [1.289–16.003]). When exposed to 2 or 3 given social hazards, the probability increases and reaches more than 9 (OR = 9.091, 95% CI = [2.260–36.560]).

The findings in relation to marital status were found to be the same as in the models explaining discrimination. Regarding males, however, no statistically significant results were found. On the other hand, married Vietnamese females had almost 6 times higher OR (compared to the reference category) (OR = 5.902, 95% CI = [1.313–26.528]).

We must also state that in addition to the above mentioned outcomes of the eight models we have computed models with the same variables but have controlled the results for a variable depicting the length of a migrants’ stay in Czechia (in years; as a continuous variable). All the results gained after adding this variable changed only marginally.

## 4. Discussion

The objectives of this study were to apply logistic regressions to a questionnaire-based immigrant study. The logistic regression models focus on a test for the individual independent variable and dependent variables (for four different sub-populations—Vietnamese, Ukrainians, females and males). All in all, the regression analysis shows us that the given independent variables (age, educational level, marital status and different types of selected social hazards in the workplace) are more important as predictors of the quality of SRH (Adj. OR) for immigrant females than males irrespective of citizenship, albeit, only for some of them and to differing extents. In all four models no statistically significant relationship was detected between poor SRH and discrimination as such (as one of the measured social hazards) at the workplace. On the other hand, in addition to confirming intersectionality regarding the unfair treatment of migrants (see also [17]) we found out that multiplicity of work exposure to the selected social hazards contributes to worsening SRH. This outcome was primarily valid for the females in our sample.

Educational level plays a role for both the Ukrainian and Vietnamese female groups in that those with a higher level of education declare poorer SRH. Perception of health seems to be negatively influenced by marital status, especially for Vietnamese females (married females perceive worse SRH).

The study indicated that social hazards in the workplace seem to be a potential social determinant of health, thereby contributing to health inequalities.

More specifically, this study, in harmony with some other research activities in Czechia [53,62], confirmed that quality of health is worse among immigrant females compared to males (see e.g., similar results in Sweden [31]). “The position of immigrant females (vis-à-vis immigrant males, e.g., Ukrainians and Vietnamese) in the host Czech society and its labour market is difficult and vulnerable to many possible forms of discrimination and threats” [62]. In addition, the worst SHR parameters were connected to females with higher and the highest education (see also the same relation to the educational level in [67]). Interestingly enough, as already mentioned, it was found out that worse SRH is linked not only to females and social hazards at workplace but also to marital status (mainly linked to married Vietnamese females)—married individuals “suffer” more than others (see [62]). All in all, one of the possible explanations is that females—just those who are often heavily burdened with their work and related unfair treatment in their rather unattractive jobs as well as all their family commitments, are under permanent stress. This leads to dissatisfaction and may further lead to mental and physical problems.

In fact, our premise touching the concept of intersectionality (gender and immigration intersect to create compounded disadvantage that impacts on health immigrants) has at least partly been detected. Intersectionality on its own contributes to poorer health. However, intersectionality [17] paired with a multiplicity of other unfair treatments, namely, multiple social hazards, has been confirmed as a significant factor in causing deterioration of perceived quality of health (see e.g., [13]).

As far as discrimination as such as one of the selected characteristics of social hazards at the workplace is concerned, we accept the premise that discrimination overlaps with the issue of ethnic discrimination. This is totally in harmony with 44 who argues: “Scientific investigations treat findings arrived at through these 2 different methods (self-reports of unfair treatment without any attribution versus those attributed to race/ethnicity) as directly comparable” ([6], p. 941).

When putting the whole story into broader Czech migratory and social contexts, two different scenarios regarding Vietnamese and Ukrainian females might exist, although they both lead to rather bad SRH. Ukrainian female’s bad SRH might also be due to the fact that (a) their work as employees in 3D (“difficult”, “dangerous” and “dirty”) positions on the Czech dual labour market is hard and stressful (their human capital often does not correspond to what they do and they are very aware of this), (b) their “permanent stopgap”—due to the often transnational, temporary, circular essence of their stay and life in Czechia, or, their stay in a new country with incomplete families and an only partly built new “second identity” lead to them missing a fully-fledged social and often family life, too. For Vietnamese females, on the other hand, just being and living in a marriage (or with other family members) and/or among their compatriots abroad with all respective commitments and burdens (also tied to their demanding family-based entrepreneurial jobs) may contribute to bad health.

## 5. Limitations

Nothwithstanding this study’s findings, there are a number of limitations that need to be acknowledged.

First, although in our esteem we worked with important independent variables which entered the models, they were rather few in number. Some, perhaps, important characteristics—such as the legal status of immigrants (regular versus irregular immigrant status), position of immigrants in different branches of the economy or on the labour market (e.g., entrepreneurs versus employees) or the extent to which they make use of intracommunity social networks could not be included into the analysis since we had no relevant information. Therefore, the impact of these and probably other confounders upon the studied association were not measured, however their role might not have been negligible. Moreover, special attention should be paid to focusing on identifying mechanisms that link discrimination to health (see e.g., [6,18]).

Second, we used the following characteristics of social risks/hazards at workplace for our analysis: discrimination at workplace (we consider it identical with racial/ethnic discrimination as such—see [16]), violence or threat of violence, time pressure or work overload and risk of accident. Nevertheless, one could question this selection and may add other variables such as e.g., workplace abuse, sexual harassment at work [16], harassment and bullying at work (inspiration in [74]), or some others. It is always a dilemma how to operationalize the given complex reality via only several selected components.

Third, the sample of respondents was gained via a quota sampling method (see above), which means that from a purely statistical point of view, the data does not allow us to generalize any results. On the other hand, we are convinced that the sampling method—as it was properly done—does not prevent our analysing the sample and getting results that in our eyes shed important light on the studied issues. Fourth, all the data was self-reported. Hence, despite any concern about the methodology, self-reporting may partly be influenced by memory and/or social desirability factors. The quantitative approach we used is one possible way to tackle the given phenomena. Of course, in order to get a more complex picture, qualitative studies should also be carried out.

## 6. Conclusions

Clearly, the health of immigrants is a topical issue and it calls for further studies tackling various aspects in this field. The results of our analysis quite clearly show how important it is to continue research activities of the studied issue—not only for scientific purposes but for improving practices and realities too. It is necessary to alert Czech (and European) politicians and administrations in order for them to start combating manifestations of various social hazards and unfair treatments more intensively, systematically and effectively and to, in general, but particularly prevent approaches which harm immigrant females. For this purpose, examining practices and policies that prevent various social hazards from being accomplished should be one of the first steps (also [12]).

## Figures and Tables

**Table 1:** Sample characteristics, workplace exposition and prevalence of not good self-rated health (N = 669).

Characteristics	Males				Females				Total		Prevalence—Fair/Poor Health (%)				
	Ukrainians		Vietnamese		Ukrainians		Vietnamese				Males		Females		Total
	N	%	N	%	N	%	N	%	N	%	Ukrain.	Vietn.	Ukrain.	Vietn.	
***Socio-demographic characteristics***															
***Age groups***															
18–30	51	23.5%	58	32.8%	54	36.2%	54	42.9%	217	32.4%	5.9%	8.6%	13.0%	7.4%	8.8%
31–40	67	30.9%	43	24.3%	41	27.5%	29	23.0%	180	26.9%	10.4%	11.6%	12.2%	31.0%	14.4%
41–62	99	45.6%	76	42.9%	54	36.2%	43	34.1%	272	40.7%	16.2%	21.1%	29.6%	34.9%	23.2%
***Education level***															
Primary	102	47.0%	103	58.2%	62	41.6%	67	53.2%	334	49.9%	14.7%	12.6%	14.5%	16.4%	14.4%
Secondary	78	35.9%	58	32.8%	63	35.6%	44	34.9%	233	34.8%	9.0%	15.5%	11.3%	20.5%	13.3%
University	37	17.1%	16	9.0%	34	22.8%	15	11.9%	102	15.2%	10.8%	25.0%	38.2%	53.3%	28.4%
***Marital status***															
Married	109	50.7%	112	63.3%	74	49.7%	73	57.9%	368	55.2%	15.6%	19.6%	24.3%	34.2%	22.3%
Others	106	49.3%	65	36.7%	75	50.3%	53	42.1%	299	44.8%	8.5%	6.2%	13.3%	5.7%	8.7%
***Social hazards in the workplace***															
***Discriminations***															
Not exp.	143	65.9%	100	56.5%	94	63.1%	87	69.0%	424	63.4%	9.1%	18.0%	19.1%	18.4%	15.3%
Exposed	74	34.1%	77	43.5%	55	36.9%	39	31.0%	245	36.6%	17.6%	10.4%	18.2%	30.8%	17.6%
***Violence or threat of violence***															
Not exp.	181	83.4%	131	74.0%	139	93.3%	107	84.9%	558	83.4%	11.0%	14.5%	18.7%	21.5%	15.8%
Exposed	36	16.6%	46	26.0%	10	6.7%	19	15.1%	111	16.6%	16.7%	15.2%	20.0%	26.3%	18.0%
***Time pressure or overload of work***															
Not exp.	97	44.7%	86	48.6%	64	43.0%	69	54.8%	316	47.2%	8.2%	15.1%	6.3%	17.4%	11.7%
Exposed	120	55.3%	91	51.4%	85	57.0%	57	45.2%	353	52.8%	15.0%	14.3%	28.2%	28.1%	20.1%
***Risk of accident***															
Not exp.	111	51.2%	130	73.4%	116	77.9%	108	85.7%	465	69.5%	8.1%	15.4%	16.4%	20.4%	15.1%
Exposed	106	48.8%	47	26.6%	33	22.1%	18	14.3%	204	30.5%	16.0%	12.8%	27.3%	33.3%	18.6%
**Total**	217	100%	177	100%	149	100%	126	100%	669	100%	12.0%	14.7%	18.8%	22.2%	16.1%

Note: SRH was dichotomized as “Good” (Good/very good) or “Not good” (Poor/poor/not good-not bad). Education level was categorized into three groups: Primary − basic + secondary without graduation; Secondary − secondary with graduation; University − post-secondary education + university. Source: own research.

**Table 2:** The association of SRH, workplace loads and demographic variables, Ukrainians, Vietnamese, males and females (Dependent variable: 1 = Not good SRH; 0 = Good SRH; Adj. odds ratio OR and 95 % CI, 4 log. models).

Characteristics	Adj. OR	95% C.I.		Adj. OR	95% C.I.		Adj. OR	95% C.I.		Adj. OR	95% C.I.	
	Ukrainian Males			Ukrainian Females			Vietnamese Males			Vietnamese Females		
	Model 1			Model 2			Model 3			Model 4		
**Education level**												
Primary	1.443	0.431	4.832	**0.280**	0.101	0.778	**0.583**	0.152	2.239	**0.169**	0.043	0.667
Secondary	0.741	0.196	2.803	**0.239**	0.077	0.745	**0.617**	0.197	3.395	0.336	0.080	1.410
University (REF.)	1			1			1			1		
**Marital status**												
Others (REF.)	1			1			1			1		
Married	**1.595**	0.611	4..164	1.960	0.784	4.898	1.950	0.552	6.884	3.381	0.826	13.840
**Discrimination**												
Not exposed (REF.)	1			1			1			1		
Exposed by 1 factor	2.137	0.913	4.999	0.980	0.395	2.432	0.502	0.197	1.276	2.911	0.999	8.483
**Age (contin.)**	1.033	0.982	1.087	1.032	0.982	1.085	1.054	0.998	1.112	1.093	1.022	1.168
	Model 5			Model 6			Model 7			Model 8		
**Education level**												
Primary	1.169	0.338	4.044	**0.227**	0.074	0.698	0.523	0.137	2.001	**0.107**	0.025	0.459
Secondary	0.681	0.177	2.615	**0.180**	0.051	0.631	0.729	0.179	2.965	**0.221**	0.050	0.968
University (REF.)	1			1			1			1		
**Marital status**												
Others (REF.)	1			1			1			1		
Married	1.667	0.638	4.357	2.021	0.775	5.266	2.132	0.601	7.561	**5.902**	1.313	26.528
**Multiple forms of social hazards**												
Not exposed (REF.)	1			1			1			1		
Exposed by 1 factor	2.662	0.661	10.727	**4.541**	1.289	16.003	0.869	0.316	2.391	0.725	0.228	2.300
Exposed by 2 factors	**3.919**	1.226	12.528	**9.091**	2.260	36.560	1.060	0.351	3.204	**5.239**	1.339	20.496
**Age (contin.)**	1.037	0.985	1.092	1.040	0.987	1.097	1.048	0.994	1.105	1.069	1.002	1.141

Notes: The reference frame is university education level, not married marital status and not exposed to workplace loads. Grey boxes are significant. Education level was categorized into three groups: Primary − basic + secondary without graduation; Secondary − secondary with graduation; University—post-secondary education + university. Bold Adj. OR when *p* < 0.05. Source: own research.
